# Supplementary material for: Evidence of p75 Neurotrophin Receptor Involvement in the Central Nervous System Pathogenesis of Classical Scrapie in Sheep and a Transgenic Mouse Model
Source: Int J Mol Sci. 2021 Mar 8;22(5):2714. doi: 10.3390/ijms22052714 (PMC7962525; doi:10.3390/ijms22052714)
Supplement: Supplementary file 1 [file ijms-22-02714-s001.pdf]

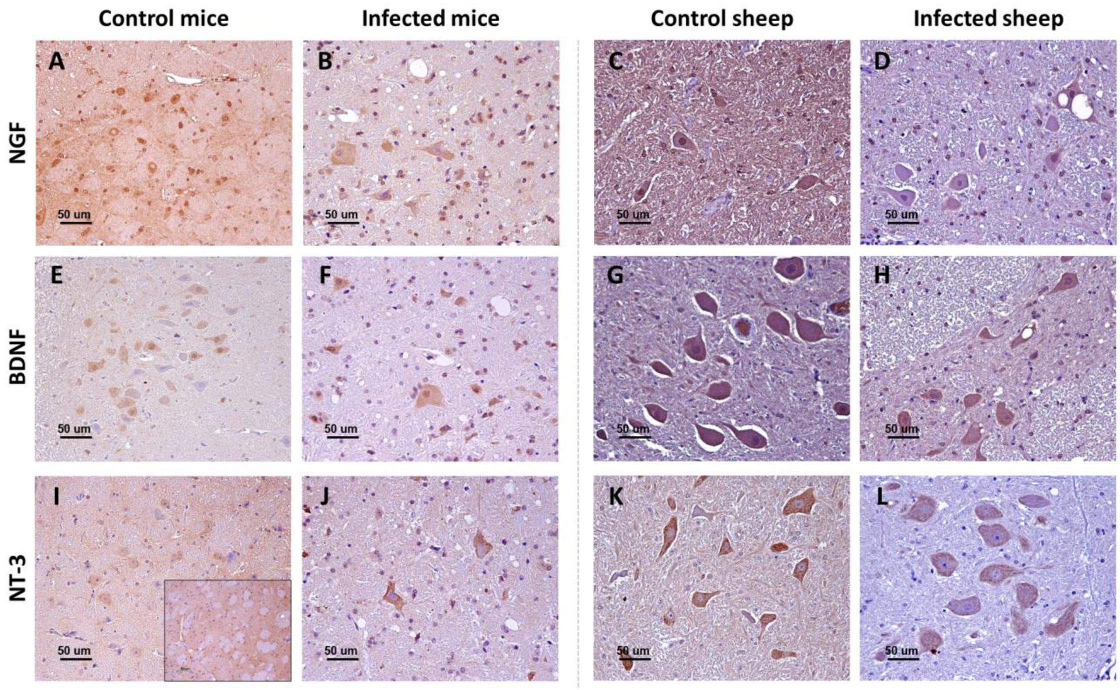

Supplementary Figure S1. Neurotrophins in the brain of infected and control mice and sheep. All microphotographs (A-L) were taken from medulla oblongata, and I [inset] was taken from striatum.

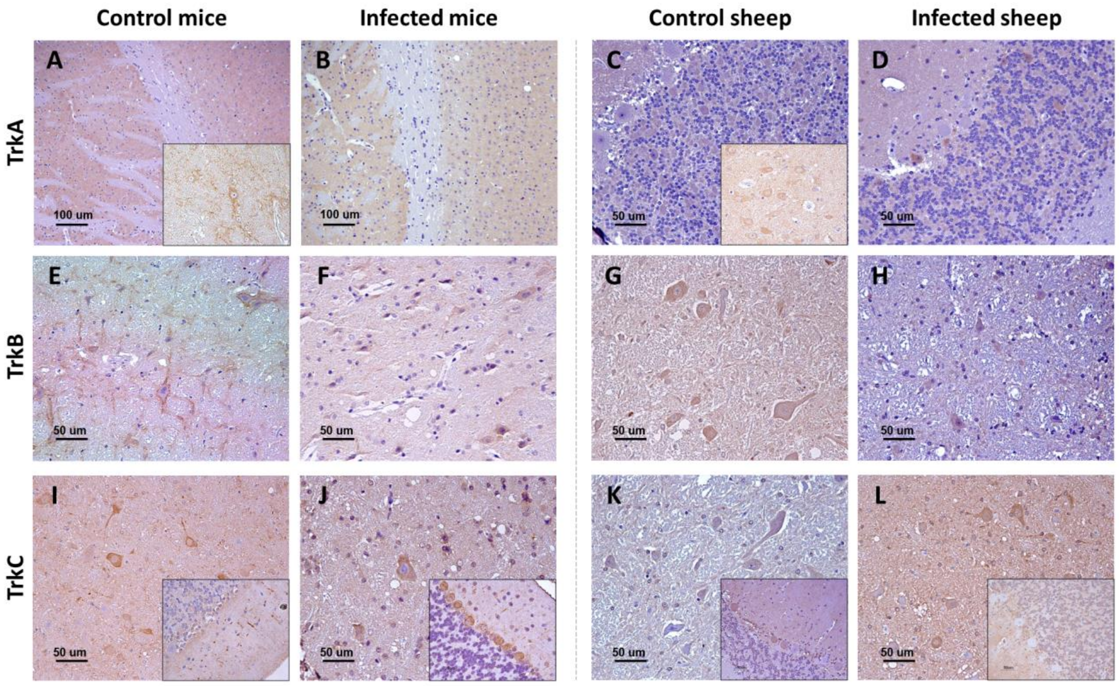

Supplementary Figure S2. Neurotrophin receptors in the brain of infected and control mice and sheep. Microphotographs taken from frontal cortex/striatum (A-B), medulla oblongata (E-P and A [inset]), cerebellar cortex (C-D and I-L [insets]), hippocampus (C[inset] and O [inset]) and corpus callosum (M [inset]).

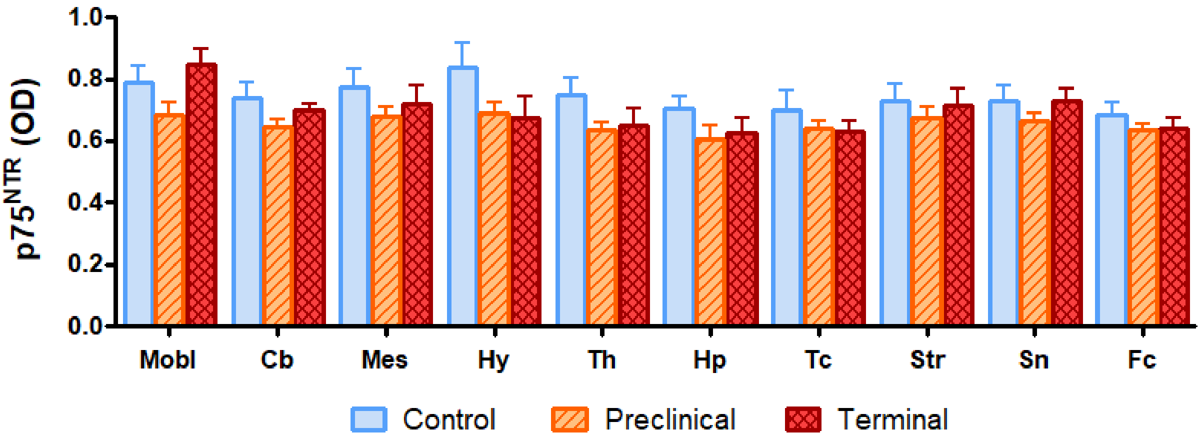

Supplementary Figure S3. Image analysis of global p75^{NTR} immunostaining in mice brains. No significant differences between infected, preclinical and terminal mice were noted in any brain area. Mobl: medulla oblongata, Cb: cerebellar cortex, Mes: mesencephalon, Hy: hypothalamus, Th: thalamus, Hp: hippocampus, Tc: cerebral cortex at the level of thalamus, Str: striatum, Sn: septal nuclei, Fc: frontal cortex. OD: optical density. Error bars represent SEM.

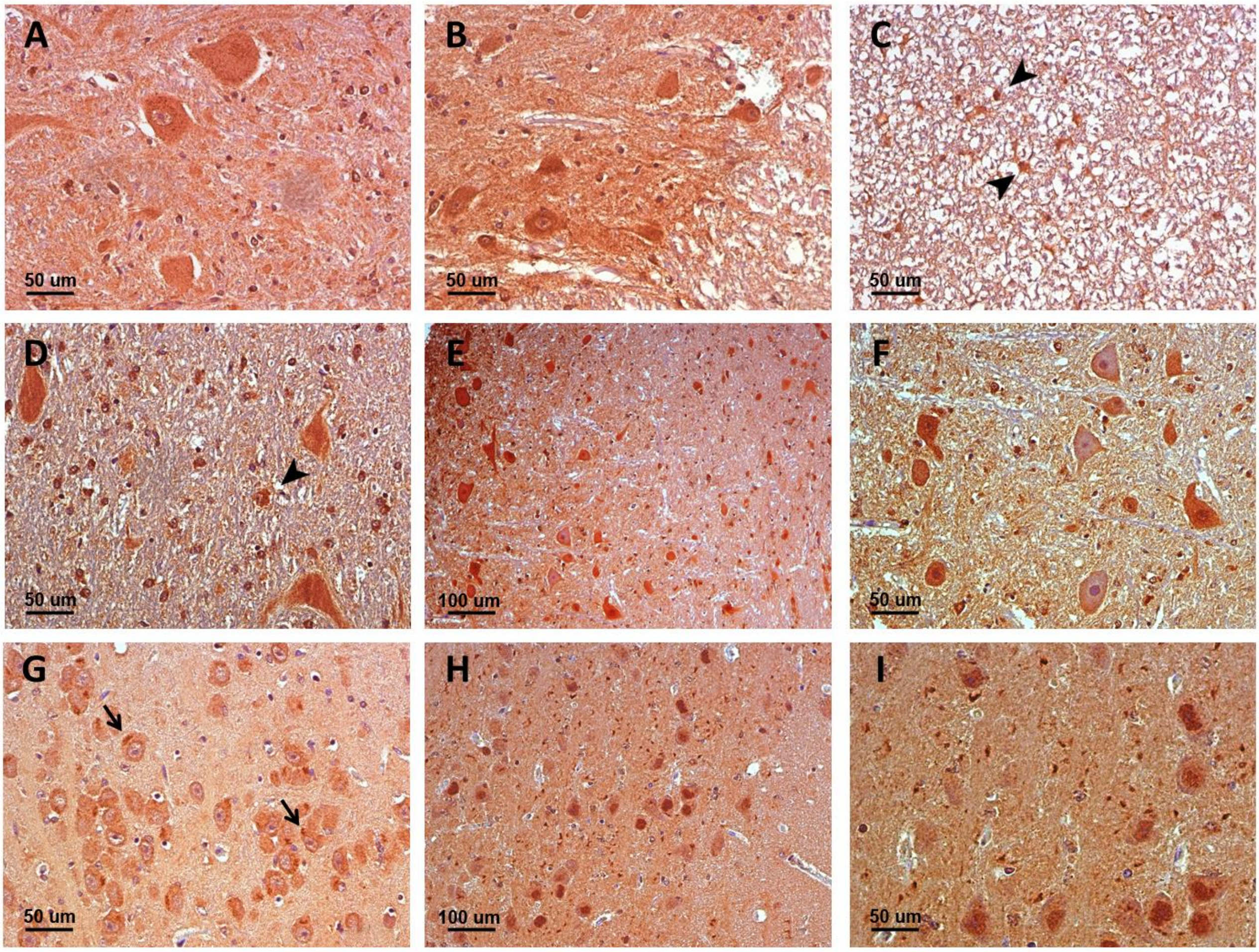

Supplementary Figure S4. Immunohistochemistry for p75^{NTR} in brain of a healthy control sheep (A-F) and a naturally scrapie-infected sheep at terminal stage (G-J). (A, B) Granular intraneuronal cytoplasmic staining in motor neurons in ventral horns of cervical spinal cord. (C) Intraglial cytoplasmic staining (*arrowhead*) in the white matter of cervical spinal cord. (D) Intraneuronal and intraglial (*arrowhead*) cytoplasmic staining in pons. (E, F) Granular intraneuronal cytoplasmic staining and neuropil punctuate deposits in oculomotor nucleus of the mesencephalon. (G) Granular intracytoplasmic deposits (*arrows*) in pyramidal neurons of the CA3 region of hippocampus. (H, I) Coarse particulate deposits in neuropil in CA3 region of hippocampus.

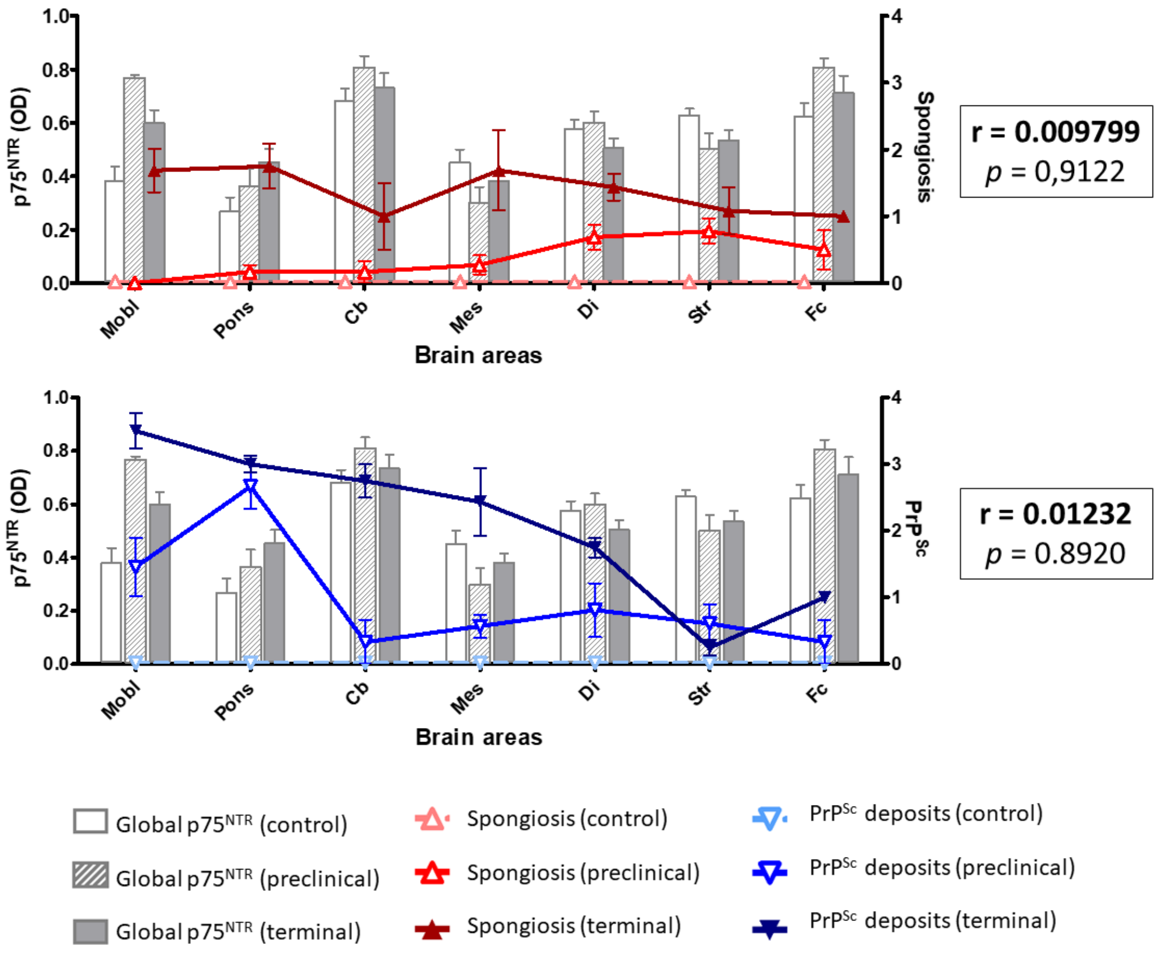

Supplementary Figure S5. Distribution of spongiform change (A) and PrP^{Sc} deposits (B) in comparison with the distribution of global $p75^{NTR}$ labeling in the brain of control, preclinical and terminal sheep. Spearman's correlation analyses showed low correlation coefficients (r) that were not statistically significant. Mobl: medulla oblongata, Pons: pons, Cb: cerebellar cortex, Mes: mesencephalon, Di: diencephalon, Str: striatum, Fc: frontal cortex. OD: optical density. Error bars represent SEM.

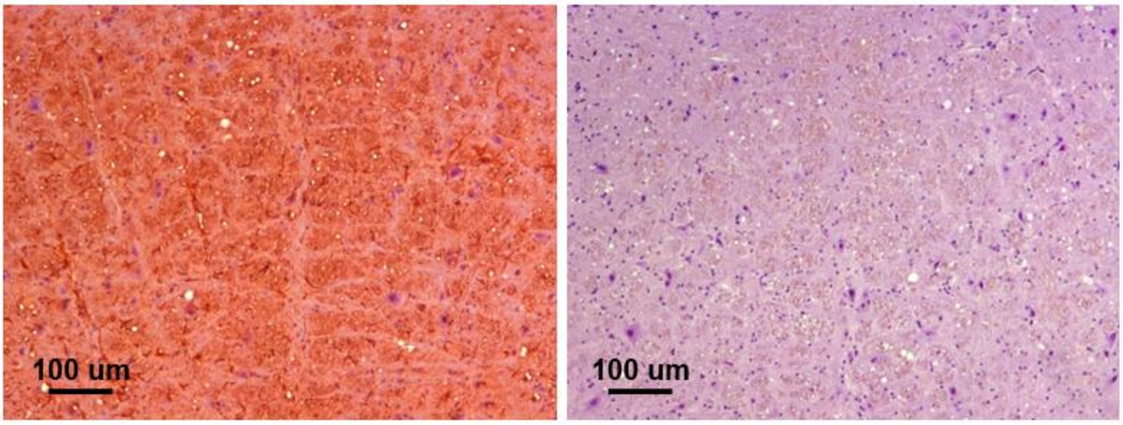

Supplementary Figure S6. Comparison of a p75^{NTR}-immunolabelled slide (A) versus a immunohistochemistry performed skipping incubation with the primary antibody (B), demonstrating the absence of background signal.

Supplementary Table S1. Statistical comparison between terminal, preclinical and control mice for the semi-quantitative parameters evaluated (glial $p75^{NTR}$ labeling, spongiosis, PrP^{Sc} deposits and gliosis).

	Glial $p75^{NTR}$ labeling							
	Mean			*p* -value (Kruskal-Wallis)		Dunn's post-hoc test		
	Control	Preclinical	Terminal			Control *vs* Preclinical	Control *vs* Terminal	Preclinical *vs* Terminal
**Mobl**	1,50	1,92	3,80	**0,0187**	*	ns	*	ns
**Cb**	0,00	0,08	0,00	> 0,05	ns	ns	ns	ns
**Mes**	0,58	1,00	2,80	**0,0067**	**	ns	**	ns
**Hy**	0,67	0,67	1,40	> 0,05	ns	ns	ns	ns
**Th**	1,67	1,08	1,60	> 0,05	ns	ns	ns	ns
**Hp**	1,83	1,33	1,40	> 0,05	ns	ns	ns	ns
**Tc**	0,83	0,92	1,00	> 0,05	ns	ns	ns	ns
**Str**	1,67	0,58	2,80	**0,0020**	*	ns	ns	ns
**Sn**	0,83	1,50	1,00	> 0,05	ns	ns	ns	ns
**Fc**	1,17	0,50	1,20	> 0,05	ns	ns	ns	ns

	PrP^{Sc} deposits							
	Mean			*p* -value (Kruskal-Wallis)		Dunn's post-hoc test		
	Control	Preclinical	Terminal			Control *vs* Preclinical	Control *vs* Terminal	Preclinical *vs* Terminal
**Mobl**	0,00	2,75	3,33	**0,0021**	**	*	**	ns
**Cb**	0,00	0,42	0,50	> 0,05	ns	ns	ns	ns
**Mes**	0,00	1,67	3,33	**0,0036**	**	ns	**	ns
**Hy**	0,00	1,83	2,50	**0,0279**	*	ns	*	ns
**Th**	0,00	2,08	2,20	**0,0074**	**	*	*	ns
**Hp**	0,00	1,00	0,20	> 0,05	ns	ns	ns	ns
**Tc**	0,00	0,50	1,30	**0,0293**	*	ns	*	ns
**Str**	0,00	1,42	2,50	**0,0117**	**	ns	**	ns
**Sn**	0,00	1,50	2,33	**0,0031**	**	ns	**	ns
**Fc**	0,00	0,70	1,58	**0,0040**	**	ns	**	ns

	Spongiosis							
	Mean			*p* -value (Kruskal-Wallis)		Dunn's post-hoc test		
	Control	Preclinical	Terminal			Control *vs* Preclinical	Control *vs* Terminal	Preclinical *vs* Terminal
**Mobl**	1,33	1,67	3,70	**0,0081**	**	ns	*	*
**Cb**	0,42	0,08	0,20	> 0,05	ns	ns	ns	ns
**Mes**	1,00	1,25	3,15	**0,0058**	**	ns	**	*
**Hy**	0,25	0,50	2,40	**0,0063**	**	ns	**	*
**Th**	1,17	1,00	2,90	**0,0046**	**	ns	*	**
**Hp**	0,92	0,33	1,60	**0,0223**	*	ns	ns	*
**Tc**	1,33	0,75	2,10	**0,0028**	**	ns	ns	**
**Str**	1,42	0,58	2,70	**0,0017**	**	ns	ns	**
**Sn**	0,58	0,50	1,80	**0,0387**	*	ns	ns	ns
**Fc**	1,25	0,42	1,70	**0,0066**	**	**	ns	**

	Gliosis							
	Mean			*p* -value (Kruskal-Wallis)		Dunn's post-hoc test		
	Control	Preclinical	Terminal			Control *vs* Preclinical	Control *vs* Terminal	Preclinical *vs* Terminal
**Mobl**	1,30	2,25	3,75	**0,0016**	**	ns	**	ns
**Cb**	0,90	0,58	0,75	> 0,05	ns	ns	ns	ns
**Mes**	1,30	1,83	3,25	**0,0017**	**	ns	**	*
**Hy**	0,80	1,00	2,25	**0,0025**	**	ns	**	*
**Th**	1,40	1,50	1,92	> 0,05	ns	ns	ns	ns
**Hp**	1,80	1,50	2,00	> 0,05	ns	ns	ns	ns
**Tc**	1,20	1,00	1,50	> 0,05	ns	ns	ns	ns
**Str**	1,90	0,50	2,50	**0,0230**	*	ns	ns	*
**Sn**	1,50	1,58	2,58	**0,0025**	**	ns	ns	**
**Fc**	1,50	1,00	1,50	> 0,05	ns	ns	ns	ns

Supplementary Table S2. Statistical comparison between terminal, preclinical and control sheep for the semi-quantitative parameters evaluated (spongiosis and PrP^{Sc} deposits and gliosis).

	**Spongiosis**							
	**Mean**			***p*-value (Kruskal-Wallis test)**		**Dunn's post-hoc test**		
	**Control**	**Preclinical**	**Terminal**			**Control *vs* Preclinical**	**Control *vs* Terminal**	**Preclinical *vs* Terminal**
**Mobl**	0,00	0,00	1,69	**< 0,0001**	***	ns	***	***
**Pons**	0,00	0,17	1,75	**0,0002**	***	ns	***	*
**Cb**	0,00	0,17	1,00	> 0,05	ns	ns	ns	ns
**Mes**	0,00	0,27	1,69	**0,0003**	***	ns	***	*
**Thal**	0,00	0,69	1,44	**< 0,0001**	***	*	***	ns
**Str**	0,00	0,78	1,08	**0,0010**	***	**	**	ns
**Fc**	0,00	0,50	1,00	> 0,05	ns	ns	ns	ns

	**PrP^{Sc} deposits**							
	**Mean**			***p*-value (Kruskal-Wallis test)**		**Dunn's post-hoc test**		
	**Control**	**Preclinical**	**Terminal**			**Control *vs* Preclinical**	**Control *vs* Terminal**	**Preclinical *vs* Terminal**
**Mobl**	0,00	1,46	3,50	**< 0,0001**	***	*	***	ns
**Pons**	0,00	2,67	3,00	**0,0004**	***	*	***	ns
**Cb**	0,00	0,33	2,75	> 0,05	ns	ns	ns	ns
**Mes**	0,00	0,57	2,44	**< 0,0001**	***	ns	***	ns
**Thal**	0,00	0,81	1,75	**0,0009**	***	ns	**	ns
**Str**	0,00	0,61	0,25	> 0,05	ns	ns	ns	ns
**Fc**	0,00	0,33	1,00	> 0,05	ns	ns	ns	ns
